# Effects of liquid smoke preparations on shelf life and growth of wild type mold and *Aspergillus flavus* in a model semi moist pet food

**DOI:** 10.3389/fmicb.2023.1154765

**Published:** 2023-04-20

**Authors:** Aiswariya Deliephan, Janak Dhakal, Bhadriraju Subramanyam, Charles G. Aldrich

**Affiliations:** ^1^Department of Grain Science and Industry, Kansas State University, Manhattan, KS, United States; ^2^Department of Agriculture, Food and Resource Sciences, University of Maryland Eastern Shore, Princess Anne, MD, United States

**Keywords:** antifungal, foodborne fungi, clean labeling, preservative, liquid smoke, pet food, intermediate moisture food, mold

## Abstract

Liquid smoke is a naturally derived flavor component and preservative with known antimicrobial properties. To our knowledge, there is a paucity of information on antifungal potential of liquid smoke against toxigenic fungi like *Aspergillus flavus* that produce mycotoxins in human and pet foods. Semi-moist pet food with high moisture content (20–30%) is susceptible to mold contamination and requires intervention. The objectives of this study were to determine the effects of liquid smoke preparations on the growth of wild-type mold and *A. flavus* in semi-moist pet food. Semi-moist pet food was formulated with eight different liquid smoke preparations (S1–S8) containing varying amounts of organic acids, phenol and carbonyl compounds (ranging from low to high) at 0% (untreated), 0.5, 1, 2, and 4% (w/w). A positive control consisted of 0.2% potassium sorbate known to inhibit mold growth. Shelf life was estimated by storing the samples at 28°C and 65–70% RH over 30 days and recording the number of days until the appearance of visible wild-type mold. In another experiment, samples were spot inoculated with *A. flavus* (∼10,000 CFU/mL), incubated at 25°C, and analyzed for fungal growth at sampling intervals of 2 days over a 35-day period. Liquid smoke at 0.5, 1, 2, and 4% extended the shelf life of samples on an average by a total of 11.6, 12.5, 17.2, and 24.1 days when compared to the untreated samples (7.7 days). The smoke preparations Cloud S-C100 (S3) and Code-10 (S6) (high carbonyl, medium/low phenol) were the most effective (*P* < 0.05) in prolonging the number of days to visible mold growth (26–28 days). In the challenge study with *A. flavus*, Cloud S-C100 (S3), Cloud S-AC15 (S8) (high to medium carbonyl, low phenol), and Code 10 (S6) (base smoke) reduced (*P* < 0.05) mold counts by 1.0, 1.7, and 2.5 logs when compared to the untreated samples at 1, 2, and 4%, respectively. Addition of smoke at 0.5% did not reduce mold counts. The carbonyl preparations of liquid smoke were the most effective at enhancing shelf life of semi-moist pet food, and at inhibiting *A. flavus* growth.

## 1. Introduction

One of the risk factors for human and animal food safety is the presence of toxigenic fungi and the potential for mycotoxins that they may produce. During manufacturing, foods can be contaminated with mold spores from the environment, and equipment surfaces within the facility (Deliephan et al., 2023a), especially when cereal grains are ground and the foods are pelleted or formed (Almeida et al., 2011). The potential for cross-contamination also exists from infected to non-infected ingredients within the facility (Huss et al., 2015). Mold spores prevalent in the environment contaminate packaged foods that are opened by the consumer and may amplify during food storage. Mold growth in foods ultimately causes spoilage leading to food waste. Moldy foods also reduce nutritional value (Bluma et al., 2008).

Mycotoxins can be produced by toxigenic fungi during pre-harvest (not only due to plant stress as some of them act as endophyte and produce mycotoxins, while others are pathogenic, and some mycotoxins can be related to pathogenicity–e.g., deoxynivalenol), post-harvest, storage, and transportation. They can be compounded with improper storage, and the process of cooking does not reduce their content (Cinar and Onbasi, 2019). These toxins with chemically diverse structures have been involved in disease outbreaks which have affected both animal and human health (Beardall and Miller, 1994; Prelusky et al., 1994; Deliephan et al., 2023b). *Aspergillus flavus* and *Fusarium graminearum* are toxigenic grain molds that produce the mycotoxins aflatoxin and deoxynivalenol, respectively. *A. flavus* is a pathogenic mold with a cosmopolitan distribution and known for its colonization of cereal grains, legumes, and tree nuts. *F. graminearum* is mainly found during pre-harvest and considered one of the main pathogens of small grain cereals.

Semi-moist pet food products contain meat, starch-based ingredients such as grains, and fats/oils. They are sold at a moisture content of approximately 25–35%. This makes them conducive to mold growth during storage if water activity is left uncontrolled. Thus, an intervention strategy is needed to retard food spoilage and potential food safety concerns (Zicker, 2008). The primary method to control water activity is to use humectants, polyols or sugar alcohols like propylene glycol and glycerin. Glycerin is considered a natural ingredient while propylene glycol is synthetic. Propylene glycol apart from controlling water activity is also effective at eliminating infestation of mites in semi-moist foods, whereas glycerin is the preferred natural humectant, but it can attract mites (Zhao et al., 2016). Therefore, to augment control of mold growth even when water activity is controlled, a mold inhibitor such as potassium sorbate is typically included. Potassium sorbate is synthetic, and a natural alternative is desired for “clean labeling” of pet foods in accordance with the current consumer preference trend.

Liquid smoke is a naturally derived flavor component and preservative used in human and pet foods, with known antimicrobial properties (Maga, 1988; Sunen et al., 2003; Lingbeck et al., 2014). The commercial production of liquid smoke involves pyrolysis – a process in which thermal decomposition of wood is carried out at 400–500°C in retorts or rotary ovens under the absence of oxygen. The resulting smoke is captured after a water spray separates the light and heavy fractions by gravity (Borys, 2004). The liquid smoke flavorings are usually obtained by further fractionating the resulting condensate through sequential extraction or liquid-liquid partitioning and/or solid-phase extraction technique based on the polarity and acidity of the constituents. Liquid smoke preparations in the food processing industry are used as flavoring agents, browning colorants, antioxidants, food texture enhancers and as antimicrobial agents (Maga, 1988; Coronado et al., 2002; Sunen et al., 2003; Borys, 2004).

Liquid smoke and its fractions containing phenols, carbonyls and organic compounds have been found to be effective against pathogenic bacteria like *Listeria monocytogenes* and *Staphylococcus aureus* (Sunen et al., 2003; Lingbeck et al., 2014) in meat and fish products. Studies evaluating their effects against fungi in food substrates have been limited. The use of liquid smoke to impregnate fibrous cellulosic food/meat casings to prevent mold growth was found to be effective by Chiu (1983) (U.S. Patent 4377187). Wendorff et al. (1993) evaluated the effects of liquid smoke on the growth of *Aspergillus oryzae*, *Penicillium camemberti*, and *Penicillium roqueforti* on cheddar cheese. They found that liquid smoke applied to the surface of cheese inhibited growth of *A. oryzae and* increased the lag period of *P. camemberti* and *P. roqueforti.* Milly et al. (2005) evaluated the phenol and carbonyl fractions of liquid smoke in nutrient broth against the mold *A. niger* and reported their minimum inhibitory concentrations to be in the range of 1.5 to 5%. Beyond these we could not find any available scientific literature on the antimycotic potential of liquid smoke in food matrices against grain molds such as *Aspergillus* and *Fusarium* spp. which produce mycotoxins in food and feed. Further, mold spoilage is visible and undesirable by consumers resulting in food waste.

The objectives of this research study were: (i) to investigate the effects of liquid smoke preparations on grain fungi, *Aspergillus flavus* and *Fusarium graminearum*, by determining their minimum inhibitory concentration (MIC) and minimum fungicidal concentration (MFC) in nutrient broth; (ii) to determine the number of days to observe wild-type mold growth in semi-moist pet food prepared with liquid smoke inclusions at various concentrations using a “days-to-mold” shelf-life study; and (iii) to evaluate the effects of liquid smoke preparations on the growth of *A. flavus* in semi-moist pet food using a mold challenge study.

## 2. Materials and methods

### 2.1. Fungal cultures

*Aspergillus flavus* (ATCC 15548) and *Fusarium graminearum* (ATCC 56091) procured from the American Type Culture Collection (ATCC, Manassas, VA, United States) were maintained in potato dextrose broth (PDB)-glycerol (7:3) at −80°C. Before use, the frozen stock cultures were thawed and streaked on potato dextrose agar (PDA; Difco Laboratories, Sparks, MD, USA) plates and incubated at 28°C for 72 h. Chloramphenicol (75 mg/L) was added to PDA to prevent the growth of bacteria. The fungal spores were collected from the grown culture on PDA by adding 5 mL of potato dextrose broth (PDB; Difco Laboratories, Sparks, MD, USA) to the surface of the dish. The spores were then dislodged from the solid medium using an L-shaped plastic rod. The spore suspension in nutrient broth was then collected and stored at 4°C and used as the fungal inoculum when needed.

### 2.2. Preparation of semi-moist pet food and source of liquid smoke

The modified formula of semi-moist pet food used for this study is shown in Table 1. All the ingredients were weighed according to the formula to produce 2 kg of pet food per batch. A 3.3 L planetary mixer (KitchenAid Portable Appliances, St. Joseph, MI, USA) was used to mix the ingredients at a speed of 50 rpm for 10 min. The dry ingredients were mixed first, followed by the liquid ingredients. The mixture was spread in a uniform layer of approximately 1 cm thickness on a baking tray lined with parchment paper. It was baked in a convection oven (MEA 21-93-E; Garland Commercial Industries, PA, USA) at 175°C for 10 min. The baked pet food was then cooled on a wire rack to room temperature. It was then cut into uniform cubes of size 3 × 3 × 1 cm using a stainless-steel knife.

**TABLE 1:** Formula used for manufacturing the model semi-moist pet food.

Ingredient	Percent % w/w
Water	18.0
Corn	12.5
Chicken by-product meal	12.5
Corn gluten meal	12.5
Glycerin	12.5
Wheat flour	5.8
Choice white grease	5.8
Corn syrup	5.0
Gelatin	2.5
Rice flour	4.3
Soybean meal	4.3
Molasses	1.0
Dry dog digest (flavor)	0.5
Salt	0.5
Dicalcium phosphate	1.4
Vitamin premix	0.2
Potassium chloride	0.2
Lysine hydrochloride	0.1
Trace mineral premix	0.1
Calcium carbonate	0.1
Choline chloride	0.1
Natural antioxidant	0.1
Total	100.0

Eight liquid smoke preparations S1-S8 (Table 2; Kerry Ingredients, Beloit, WI, USA) were evaluated at 0.5, 1, 2 and 4% w/w inclusion in semi-moist pet food in this study. The initial moisture content of the pet food samples was 25% dry basis. Water activity (a_*w*_) was recorded using a water activity meter (Decagon CX-2; Meter Group, Pullman, WA, USA) and pH was measured using a pH meter (P/N 54 × 002608; Oakton Instruments, Vernon Hills, IL, USA). The a_*w*_ and pH values were reported in Table 3. Semi-moist pet food made with 0.2% potassium sorbate and containing no liquid smoke served as the positive control, which is known to inhibit mold growth. Semi-moist pet food made with 0% liquid smoke and no potassium sorbate served as the “untreated” control.

**TABLE 2:** Liquid smoke preparations evaluated in the study.

Smoke preparation	Name	Description
S1	P-1720	Buffered low phenol smoke, medium carbonyl
S2	Cloud S-5	Buffered pH, low acid, low carbonyl, no phenol
S3	Cloud S-C100	Carbonyl preparation: high carbonyl, low acid, very low phenol
S4	Black deli	Basic pH smoke, zero carbonyls, organic acid salts, phenols
S5	Hickory OS-1473	Phenol preparation: high phenol, low acid, no carbonyl
S6	Code 10	Base smoke: organic acid/carbonyls/phenols
S7	Code V	Organic acid preparation: low pH, medium acid, medium carbonyl, low phenol
S8	Cloud S-AC15	High buffered organic acid + medium carbonyl preparation

**TABLE 3:** Water activity (a_w_) and pH of semi-moist pet food treatments without and with inclusion of liquid smoke (0.5–4%, w/w).

Treatment	a_w_	pH (0.5–4% liquid smoke)
Untreated	0.75–0.76	5.2–5.3
P-1720	0.75	4.6–5.3
Cloud S-5	0.75	4.9–5.2
Cloud S-C100	0.76–0.77	4.8–5.4
Black deli	0.75	4.9–5.3
Hickory OS-1473	0.75	4.6–5.3
Code 10	0.75	4.9–5.2
Code V	0.75	4.5–5.0
Cloud S-AC15	0.75	4.9–5.2

### 2.3. Minimum inhibitory concentration (MIC) and minimum fungicidal concentration (MFC)

The minimum inhibitory concentrations (MIC) of the liquid smoke treatments were determined by the broth micro- and macro-dilution assay according to the antimicrobial susceptibility testing methods described by the Clinical and Laboratory Standards Institute (Clinical and Laboratory Standards Institute [CLSI], 2014). To determine MIC, a 200 μL volume of liquid smoke preparation (Table 2) consisting of twice the desired final concentration were dispensed in the first well of a 96-well microtiter plate and 100 μL of sterile water in rest of the wells. A serial two-fold dilution of the liquid smoke preparations was performed starting from 50 to 0.05% v/v concentration. A 100 μL aliquot of fungal (*Aspergillus flavus* or *Fusarium graminearum*) culture (∼10,000 CFU/mL) was added to each well of the plate already containing the 100 μL of decreasing concentrations of liquid smoke preparations, to make a final volume of 200 μL per well. The positive control consisted of fungal inoculum only (no treatment), and the negative control consisted of PDB alone (no treatment, no inoculum). The MIC was determined as the lowest concentration of liquid smoke that inhibited visible growth of *A. flavus* or *F. graminearum* in the microtiter plate for 30 days on incubation at 28°C. Inhibition of mold growth was visually assessed by absence of mycelial growth or turbidity in the plate wells over a period of 30 days of incubation at 28°C (Lopez-Malo et al., 2007). A similar experiment was also conducted with potassium sorbate as treatment in the place of liquid smoke to determine its MIC against *A. flavus* and *F. graminearum*. The study was replicated three times.

To determine minimum fungicidal concentration (MFC), 100 μL of sample from each well from the MIC experiment was plated on PDA for enumeration of fungal colonies at each concentration of treatment (liquid smoke or potassium sorbate) after incubation at 28°C for 72 h. MFC was determined as the lowest concentration of treatment that caused absence (≤1 colony) of fungal growth on the agar plate.

### 2.4. Days-to-mold (shelf life) study

For this study, the liquid smoke preparations (Table 2) were evaluated at 0.5, 1, 2, and 4% w/w inclusion in semi-moist pet food. Semi-moist pet food with no potassium sorbate and no smoke served as the untreated control. Semi-moist pet food made with 0.2% potassium sorbate and containing no smoke served as the positive control, known to inhibit mold growth. About 30 g of sample from each treatment was placed in a Whirl-Pak bag (Nasco, Ft. Atkinson, WI, USA) each with four pin holes, and placed in an environmental chamber at 28°C and 65–70% relative humidity in total darkness. The samples were observed every day over a period of 30 days for visible mold growth on the surface. The number of days for the first mold colony to appear on the surface of the pet food cubes for each sample was recorded. The study was replicated three times.

### 2.5. Mold challenge study using *Aspergillus flavus*

The fungal inoculum from cultures of *Aspergillus flavus* (ATCC 15548) were prepared as described earlier. A volume of 1 mL of the inoculum (∼10,000 CFU/mL) was spot inoculated on 25 g of semi-moist pet food using a pipette. The inoculum concentration (CFU/mL) was determined by plating on PDA. The initial moisture content of the pet food samples was 25% and the final moisture content post-inoculation was maintained at ∼29%. The inoculated samples were stored in Whirl-Pak bags as described earlier at 28°C and 65–70% relative humidity in an environmental chamber. They were analyzed for fungal growth at a sampling interval of 2 days over a period of 35 days (day 0, day 1, day 3, day 5 etc., up to day 35). For the fungal analysis, the treatment samples (25 g) were placed in sterile Whirl-Pak bags and mixed in 225 mL of buffered peptone water (Difco Laboratories, Sparks, MD, USA) and stomached for 2 min. The mixtures were serially diluted in 0.1% peptone water (Difco Laboratories, Sparks, MD, USA) and plated on PDA. Chloramphenicol (75 mg/L) was added to PDA to prevent the growth of bacteria. The plates were incubated at 28°C for 72 h and then the fungal colonies were counted. The fungal counts were expressed as log CFU/mL.

### 2.6. Statistical analysis

For the days-to-mold study, the mean number of days taken to grow mold for the treatments [untreated (0%) and smoke treatments (Table 2)] at concentrations 0.5, 1, 2, and 4% were subjected to two-way analysis of variance (ANOVA) using the GLIMMIX procedure of SAS version 9.3 statistical software, with means among treatments separated using Tukey’s test (*P* ≤ 0.05) (SAS Institute Inc, 2011). For the mold challenge study with *A. flavus*, the mean log reductions of mold count between day 0 and day 35 for the treatments [untreated (0%) and smoke treatments] at concentrations 0.5, 1, 2, and 4% were also subjected to two-way ANOVA, with means among treatments separated using Tukey’s test (*P* ≤ 0.05) (SAS Institute Inc, 2011). The linear model *y* = *a* + *bx* was fit to logarithmic reduction of mold counts over time (days) for the smoke treatments at 2 and 4%, where *a* is the intercept and *b* is the slope. The decimal reduction time or *D*-value (time taken for 1-log reduction of mold counts) was calculated as the negative-inverse of slope (Mazzola et al., 2003).

## 3. Results

The MICs and MFCs of liquid smoke preparations and potassium sorbate against *Aspergillus flavus* and *Fusarium graminearum* are shown in Table 4. The MICs of liquid smoke ranged from 1.6 to 6.3% for *A. flavus*, and 0.4 to 1.6% for *F. graminearum*. The MFCs of liquid smoke ranged from 3.1 to 12.5%, and 0.4 to 3.1%, for *A. flavus* and *F. graminearum*, respectively. For potassium sorbate, the MIC as well as the MFC were 0.195 and 0.098% for *A. flavus* and *F. graminearum*, respectively. Against *A. flavus*, the liquid smoke preparations Cloud S-C100, Code-10, and Cloud S-AC15 had the lowest MIC (1.56%), and P-1720, Cloud S-C100, and Cloud S-AC15 had the lowest MFC (3.125%). Against *F. graminearum*, P-1720, Cloud S-C100, Code-10, and Cloud S-AC15 had the lowest MIC (0.39%), and Cloud S-C100 and Cloud S-AC15 had the lowest MFC (0.39%). The least effective liquid smoke preparations against *A. flavus* were Cloud S-5 and Hickory OS-1473 with the highest MIC (6.25%) and MFC (12.5%). Against *F. graminearum*, the least effective liquid smoke preparations were Cloud S-5 and Black deli with the highest MIC (1.56%), and Cloud S-5 with the highest MFC (3.125%).

**TABLE 4:** Minimum inhibitory concentrations (MICs) and minimum fungicidal concentrations (MFCs) of liquid smoke preparations in nutrient broth (PDB) against *Aspergillus flavus* and *Fusarium graminearum* in comparison with potassium sorbate, a known mold inhibitor.

Treatment	*Aspergillus flavus*		*Fusarium graminearum*	
	**MIC (%)^1^**	**MFC (%)^1^**	**MIC (%)^1^**	**MFC (%)^1^**
P-1720	3.125	3.125	0.39	1.56
Cloud S-5	6.25	12.5	1.56	3.125
Cloud S-C100	1.56	3.125	0.39	0.39
Black deli	3.125	6.25	1.56	1.56
Hickory OS-1473	6.25	12.5	0.78	1.56
Code 10	1.56	6.25	0.39	1.56
Code V	3.125	6.25	0.78	1.56
Cloud S-AC15	1.56	3.125	0.39	0.39
Potassium sorbate	0.195	0.195	0.098	0.098

^1^The most recurring value of 3 replicates is reported as MIC or MFC.

A positive control consisted of fungal inoculum only (no treatment), and a negative control consisted of potato dextrose broth (PDB) alone (no treatment, no fungal inoculation).

The mean number of days to mold for the untreated and smoke treated samples are reported in Table 5. The positive control (0.2% potassium sorbate, no smoke) did not allow mold growth during the observation period of 30 days. The untreated (no smoke treatment) sample allowed mold growth in 7.7 days. The average days to grow mold for the smoke treatments at 0.5% ranged from 8.7 (Code V) to 13.3 days (Cloud S-C100 and Code 10), at 1% ranged from 9.3 (Code V) to 15.3 days (Black deli), at 2% ranged from 14.3 (Code 10 and Cloud S-5) to 21.0 days (Hickory OS-1732 and Cloud S-AC15), and at 4% ranged from 18.0 (Black deli) to 28.0 days (Cloud S-C100 and Code-10). Grouping of treatment means (Table 5) by Tukey’s *post-hoc* test indicated that among the smoke treatments, Cloud S-C100 and Cloud S-AC15 were the most effective (*P* < 0.05) in prolonging the number of days to mold detection whereas Code-V and Cloud S-5 were the least effective. Within each smoke treatment, except for Black deli, there was no difference between 0.5 and 1% concentrations (*P* > 0.05), and 4% concentration was the most effective (*P* < 0.05) in prolonging the shelf life of pet food samples.

**TABLE 5:** Mean number of days taken to observe wild-type mold growth in a model semi-moist pet food with inclusion of liquid smoke treatments at 0.5, 1, 2, and 4% in comparison with untreated (0% smoke).

Treatment	Number of days to mold (Mean ± SE)^1,2,3^				
	**Concentration (%)**				
	**0.0**	**0.5**	**1.0**	**2.0**	**4.0**
Untreated	7.7 ± 0.3^c,A^	–	–	–	–
P-1720		10.7 ± 0.7^ab,A^	11.7 ± 1.2^ab,AB^	14.5 ± 1.2^ab,B^	24.0 ± 0.0^ab,C^
Cloud S-5		12.0 ± 1.2^b,AB^	10.3 ± 0.7^b,A^	14.3 ± 0.7^b,B^	22.0 ± 1.6^b,C^
Cloud S-C100		13.3 ± 0.9^a,A^	15.0 ± 1.0^a,AB^	18.8 ± 0.8^a,B^	28.0 ± 0.0^a,C^
Black deli		10.7 ± 0.7^ab,A^	15.3 ± 2.4^ab,B^	19.7 ± 0.9^ab,C^	18.0 ± 0.0^ab,BC^
Hickory OS-1473		12.0 ± 2.1^ab,A^	13.0 ± 0.0^ab,A^	21.0 ± 1.5^ab,B^	25.0 ± 0.0^ab,C^
Code 10		13.3 ± 1.2^ab,AB^	11.0 ± 0.6^ab,A^	14.3 ± 1.2^ab,B^	17.0 ± 2.3^ab,C^
Code V		8.7 ± 0.3^b,A^	9.3 ± 0.3^b,A^	14.7 ± 1.5^b,B^	26.0 ± 1.0^b,C^
Cloud S-AC15		12.0 ± 1.0^a,A^	14.7 ± 1.8^a,A^	21.0 ± 0.8^a,B^	26.0 ± 0.8^a,C^

^1^Each mean is based on *n* = 3 replications.

^2^Means among the treatments within each concentration followed by different letters in lower case are significantly different (*P* < 0.05, by Tukey’ test).

^3^Within each treatment, means among concentrations followed by different letters in upper case are significantly different (*P* < 0.05, by Tukey test).

Potassium sorbate treatment (positive control) did not show visible mold growth during the 30-day period.

**FIGURE 1:**
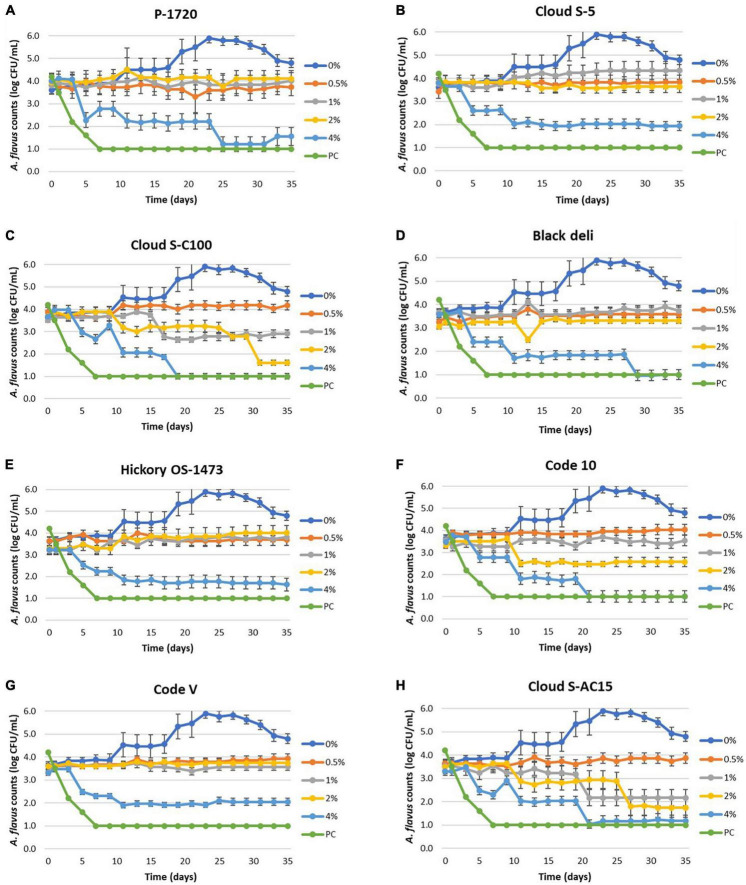
**(A–H)** Mean logarithmic counts (log CFU/mL) of *Aspergillus flavus* in semi-moist pet food treatments with inclusion of liquid smoke preparations P-1720 **(A)**, Cloud S-5 **(B)**, Cloud SC-100 **(C)**, Black deli **(D)**, Hickory OS-1473 **(E)**, Code 10 **(F)**, Code V **(G)**, and Cloud S-AC15 **(H)** at 0.5, 1, 2, and 4% concentrations in comparison with untreated (0% smoke). Potassium sorbate treatment (PC, positive control) did not show mold growth during the 35-day period. The detection limit is 1.0 log CFU/mL for sampling using PDA plates.

Reduction in mold counts of *Aspergillus flavus* was observed due to liquid smoke inclusion in the semi-moist pet food samples as shown in Figures 1A–H and Table 6. The untreated sample (0% smoke) had an increase in mold counts up to 5 logs from an initial load of 3.5 logs over the 35 days incubation period. Across all treatments, inclusion of liquid smoke at 4% decreased (*P* < 0.05) mold counts over time compared to 0, 0.5, 1, and 2%. The mean mold count log reduction for the smoke treatments, between day 0 and day 35 at 4% ranged from 1.5 logs (Code V) to 2.5 logs (Cloud S-C100), at 2% ranged from 0.2 logs (Black deli) to 1.9 logs (Cloud S-C100), and at 1% ranged from 0.6 logs (Cloud S-C100) to 1.3 logs (Cloud S-AC15). At 0.5% none of the smoke treatments had any log reduction in mold counts and instead showed an increase of 0.1 logs (Black deli) to 0.7 logs (Cloud S-C100). Also at 1%, the smoke treatments P-1720, Cloud S-5, Black deli, Hickory OS-1473, and Code-V experienced a logarithmic increase in mold counts (0.1–0.8 logs), and at 2% there was an increase of 0.1–0.6 logs for P-1720, Cloud S-5, Hickory OS-1473, and Code-V. Across the different time points, mold count reductions were observed after 16 days (*P* < 0.05) on average across the smoke treatments. Within each smoke treatment, there was no difference between 0.5 and 1% concentrations (*P* > 0.05) for P-1720, Black deli, and Hickory OS-1473, no difference between 1 and 2% concentrations (*P* > 0.05) for P-1720, Cloud S-C100, Hickory OS-1473, Code-V, and Cloud S-AC15, and 4% concentration was the most effective (*P* < 0.05) at reducing mold counts. Among the smoke treatments, Cloud S-AC15, Cloud S-C100, and Code-10 were the most effective (*P* < 0.05) in reducing mold counts, whereas P-1720 and Cloud S-5 were the least effective.

**TABLE 6:** Mean logarithmic reduction (between day 0 and day 35) of *Aspergillus flavus* counts in semi-moist pet food treatments with inclusion of liquid smoke preparations at 0.5, 1, 2, and 4% concentrations in comparison with untreated (0% smoke) over 35 days incubation period.

Treatment	Log reduction (Mean ± SE)^1,2,3,4^				
	**Concentration (%)**				
	**0.0**	**0.5**	**1.0**	**2.0**	**4.0**
Untreated	−1.3 ± 0.2^f,E^	–	–	–	–
P-1720		−0.2 ± 0.4^e,B^	−0.5 ± 0.4^e,BC^	−0.6 ± 0.4^e,C^	1.9 ± 0.3^e,A^
Cloud S-5		−0.3 ± 0.3^e,B^	−0.8 ± 0.4^e,C^	−0.1 ± 0.2^e,B^	1.6 ± 0.2^e,A^
Cloud S-C100		−0.7 ± 0.2^b,C^	0.6 ± 0.2^b,B^	1.9 ± 0.1^b,B^	2.5 ± 0.0^b,A^
Black deli		−0.1 ± 0.3^bc,C^	−0.2 ± 0.2^bc,C^	0.2 ± 0.1_bc,B_	2.4 ± 0.1^bc,A^
Hickory OS-1473		−0.2 ± 0.3^cd,B^	−0.3 ± 0.1^cd,B^	−0.5 ± 0.3^cd,B^	1.9 ± 0.3^cd,A^
Code 10		−0.5 ± 0.2^b,D^	0.0 ± 0.2^b,C^	0.9 ± 0.2^b,B^	2.4 ± 0.1^b,A^
Code V		−0.4 ± 0.2^de,C^	−0.1 ± 0.1^de,B^	−0.3 ± 0.2^de,BC^	1.5 ± 0.2^de,A^
Cloud S-AC15		−0.4 ± 0.2^a,C^	1.3 ± 0.4^a,B^	1.8 ± 0.4^a,B^	2.3 ± 0.2^a,A^

^1^Negative values of log reduction indicate increase of mold counts over the 35-day period.

^2^Each mean is based on *n* = 3 replications.

^3^Means among the treatments at each concentration followed by different letters in lower case are significantly different (*P* < 0.05, by Tukey’s test).

^4^Within each treatment, means among concentrations at each treatment followed by different letters in upper case are significantly different (*P* < 0.05, by Tukey’s test).

Initial load of *A. flavus* inoculated in the semi-moist pet food samples was 3.5 log CFU/mL. Potassium sorbate treatment (positive control) did not show mold growth during the 35-day period.

The *D*-values, which is the time in days required to record 1-log reduction of mold counts, for each treatment are shown in Table 7. At 4% concentration of smoke, the *D*-values ranged from 5 days (Cloud S-C100) to 14.3 days (Code-V), and at 2% the *D*-values ranged from 16.7 days (Code-10) to 50 days (Cloud S-5). *D*-values were not calculated for treatments showing no log reduction of mold counts over time. Among the smoke treatments, Cloud S-C100 and Cloud S-AC15 were the most effective in reducing *A. flavus* counts with the lowest *D*-values (5–9 days).

**TABLE 7:** Linear regression (linear model *y* = *a* + *bx*) parameters of logarithmic reduction of *Aspergillus flavus* counts in semi-moist pet food treatments with inclusion of liquid smoke preparations at 2 and 4% concentrations.

Treatment	Concentration (%)	Linear regression parameters			
		***a*** **± SE**	***b*** **± SE**	***R*** **^2^**	***D*** **-value^1,2^ (days)**
P-1720	2.0	4.07	0.00	0.01	–
	4.0	3.74	−0.15	0.77	6.7
Cloud S-5	2.0	3.87	−0.02	0.59	50.0
	4.0	3.26	−0.09	0.63	11.1
Cloud S-C100	2.0	4.25	−0.12	0.75	8.3
	4.0	3.88	−0.20	0.87	5.0
Black deli	2.0	3.10	0.01	0.13	–
	4.0	3.37	−0.14	0.80	7.1
Hickory OS-1473	2.0	3.25	0.05	0.83	–
	4.0	2.90	−0.08	0.67	12.5
Code 10	2.0	3.47	−0.06	0.58	16.7
	4.0	3.63	−0.18	0.88	5.6
Code V	2.0	3.63	0.01	0.41	–
	4.0	2.93	−0.07	0.48	14.3
Cloud S-AC15	2.0	3.94	−0.11	0.83	9.1
	4.0	3.30	−0.13	0.84	7.7

*a* and *b* are linear regression parameters. *a* = intercept; *b* = slope.

^1^*D*-value (−1/*b*) shows the time in days for 1-log reduction of *A. flavus* counts.

^2^*D*-values were not calculated for treatments showing no log reduction of mold counts over time.

The linear regression model *y* = *a* + *bx* was best fit for the 4% treatments with higher *R*^2^ values (>0.70) due to more pronounced log reductions of the mold counts. The model fit was poor (low *R*^2^ values) for most of the 2% smoke treatments and so *D*-values could not be obtained for several of the smoke treatments at 2% due to no consistent log reductions of mold count over time. However, it shows a fungistatic effect of the liquid smoke inhibiting the mold growth.

## 4. Discussion

*In vitro* microbial susceptibility tests such as MIC and MFC, are usually performed to evaluate the sensitivity of an organism to an antimicrobial agent such as an antibiotic or chemical preservative. From the MIC and MFC assays in this study, Cloud S-C100 and Cloud S-AC15 were found to be the most effective against both the fungal species in nutrient broth. The least effective were Cloud S-5, Black deli and Hickory OS-1473. This study points out that liquid smoke preparations containing medium to high carbonyl content (Cloud S-C100 and Cloud S-AC15) were more effective at inhibiting fungal growth (fungistatic or fungicidal) in broth dilution antimycotic susceptibility tests, while those containing low acid and buffered pH or high phenol content (Cloud S-5, Black deli and Hickory OS 1473) were the least effective. MIC and MFC of liquid smoke depend on several variables including composition and concentration of its components such as phenols, carbonyls and organic acids and the cultural conditions for the test organisms, and so comparison of results with those of other studies involving smoke is not simple. Studies concerning smoke condensates and their antimicrobial potential have been performed before (Maga, 1988; Sunen, 1988; Estrada-Munoz et al., 1998; Painter, 1998; Sunen et al., 2001, 2003). These previous studies were done using smoke preparations having a different composition than the ones used in the present study and concluded that phenols are responsible for the antimicrobial properties of smoke condensates. In this study we tested smoke preparations with a range of phenol, carbonyl, and organic acid contents to determine the effectiveness of the combination of these components as antimicrobial agents. Our MIC results (1.56–6.25%) are in accordance with the results reported by Milly et al. (2005) who determined the minimum inhibitory concentrations of phenol and carbonyl fractions of liquid smoke in nutrient broth against yeast (*Saccharomyces cerevisiae*) and mold (*A. niger*) and reported the MIC of *A. niger* to be in the range of 1.5 to 5%. The MIC and MFC assays in this study indicate that across all treatments, the mold *F. graminearum* was more susceptible to antimycotic or antifungal effects of liquid smoke than *A. flavus*. From the studies by Panwar et al. (2016) and Casquete et al. (2017) it was reported that *Aspergillus flavus* is more tolerant of fluctuations in growth conditions like temperature and pH and is a more “robust” mold than *F. graminearum*. This may explain why *Fusarium* was more prone to antimycotic effect from the liquid smoke than *Aspergillus*. This was also the reason the mold challenge experiments in this study using liquid smoke preparations in semi-moist pet food was conducted using only *A. flavus*. While comparing the MIC and MFC concentration results in this study, it can be found that the MFC is almost twice that of the MIC, and in some cases the same as MIC, across the different treatments. According to Pankey and Sabath (2004) when the ratio of minimum bactericidal concentration (MBC) to MIC is less than or equal to 4, the antimicrobial can be considered bactericidal. Since the MFC to MIC ratios of liquid smoke for *A. flavus* and *F. graminearum* in this study is in the range of 1 to 2, it can be considered as fungicidal and should be effective against these molds when applied in food systems (Kiprotich et al., 2021).

Based on the MIC and MFC results of liquid smoke preparations from this study, which ranged from 0.4 to 6.25%, semi-moist pet food was prepared with smoke inclusions at 0.5, 1, 2, and 4% (w/w), and a days-to-mold study was conducted to indicate shelf life qualitatively. The number of days until visible mold growth had been used for shelf-life indication in animal feed in few previous studies (Russell et al., 1991; Reese et al., 2000; Kung, 2005). Overall, at 4% concentration the liquid smoke preparations extended the shelf life of semi-moist pet food samples by 20 days or more, with the highest being 26–28 days as seen in Cloud S-C100 and Cloud S-AC15, however, at 0.5% the shelf life was prolonged by only ∼5 days, with the highest being 12–13 days (Cloud S-C100 and Cloud S-AC15) in comparison with untreated sample’s shelf life (7.7 days). Among the smoke treatments averaging across concentrations, Cloud S-C100 and Cloud S-AC15 containing medium to high carbonyl content were the most effective in prolonging the number of days to mold whereas Code-V (organic acid preparation of liquid smoke) and Cloud S-5 (buffered pH, low acid preparation) were the least effective. These results show that high carbonyl content in liquid smoke has a fungicidal effect on wild-type environmental mold, unlike that reported by some previous studies (Maga, 1988; Sunen, 1988; Estrada-Munoz et al., 1998; Painter, 1998; Sunen et al., 2001, 2003) which attributed the antimicrobial effect of smoke to be the phenol content. Deans and Ritchie (1987), and Dorman and Deans (2000) who studied antimicrobial activity of plant essential oils and Tayel (2010) who studied antifungal effects of smoke from smoldering medicinal plants like cinnamon on *A. flavus* also attributed the antimicrobial and antifungal properties to compounds containing phenolic groups present in the plants and their essential oils. Our results showed that carbonyl components in liquid smoke have antimycotic activity against environmental molds. The proposed mechanism of action of carbonyl compounds in liquid smoke against micro-organisms or bacterial cells was explained by Lingbeck et al. (2014). Carbonyls inhibit microbial growth by penetrating the cell wall and inactivating enzymes located in the cytoplasm and the cytoplasmic membrane (Milly et al., 2005). Carbonyls act by condensing with the free, primary amino-groups in the polypeptide chains, primarily in the side chains of basic amino-acids. These amino groups may be an essential part of active site of the enzyme, or they may function to bind the substrate by hydrogen-bonding (Painter, 1998). Even if the carbonyls cannot access the interior of a microbial cell, they can still inhibit growth by interfering with the uptake of nutrients.

The water activity of semi-moist pet foods usually ranges from 0.6 to 0.8 a_*w*_ and pH is about 5. The pH of each of the liquid smoke preparations used in this study varied considerably (ranging from acidic to alkaline) but pH of the semi-moist pet food treatments with inclusion of liquid smoke in this study ranged from 4.6 to 5.3 (Table 3). This higher range of pH is possibly due to the buffering capacity of the food matrix. So, we believe the low pH effect is not contributing substantially to the antifungal properties of liquid smoke; instead, it could be the synergistic action of carbonyls/organic acids/phenols present. It is known that the antimicrobial mode of action of organic acids is not dependent on pH alone, but also on pKa values (concentration of undissociated form of the organic acid), which explains their antimicrobial activity even at a slightly higher pH environment (Breidt et al., 2004). More studies are required to investigate the action of carbonyl compounds in liquid smoke against molds and bacteria and any synergistic effect with organic acids and phenolic compounds.

To better understand the fungicidal or fungistatic potential of liquid smoke preparations in a model food substrate, i.e., semi-moist pet food over time, a mold challenge study with *A. flavus* was conducted with liquid smoke inclusions at 0.5, 1, 2, and 4% w/w and enumerating the mold counts over a 35-day period. When comparing the initial inoculated mold counts (day 0) with the final mold counts on day 35, the log reductions for Cloud S-C100, Cloud S-AC15, and Code 10 at 4% were 2.3–2.5 logs, at 2% were 0.9–1.9 logs, at 1% were 0.6–1.3 logs, and at 0.5% no log reductions were observed (Table 6). Among the smoke treatments, Cloud S-AC15, Cloud S-C100, and Code-10 were the most effective in reducing mold counts, whereas P-1720 and Cloud S-5 were the least effective. These results indicate that high to medium carbonyl content (Cloud S-C100 and Cloud S-AC15) and base smoke (Code-10) preparations of liquid smoke had an antimycotic effect on *A. flavus* growth over time, whereas buffered pH smoke preparations like P-1720 and Cloud S-5 were the least effective against *A. flavus*. In our study, the mold counts over the 35-day incubation period of the various smoke treatments (Figure 1) indicate that even though there were no pronounced logarithmic reduction of mold counts for the lower concentrations 0.5 and 1% of smoke treatments, the *A. flavus* counts did not increase compared to the untreated samples. This shows a fungistatic effect of the smoke preparations. This result can be explained by the prolonged lag phase in the growth curves of *A. niger* when exposed to liquid smoke in the study by Milly et al. (2005). As they tested the effects of liquid smoke only in nutrient broth and not in any food substrate, our mold challenge study in model semi-moist pet food helps us in further understanding the antimycotic effects of applied liquid smoke. Milly et al. (2005) reported that high carbonyl and low pH fractions of liquid smoke were effective at inhibiting or prolonging lag phase in the growth curves of the tested micro-organisms including *A. niger.* Low pH effect of liquid smoke could not be correlated with the antimycotic effect in our study because even though the liquid smoke may have acidic pH, the pH in the semi-moist food substrate appeared to have been buffered due to the presence of proteins and starches (Table 3).

The *D*-values (Table 7) for *A. flavus* exposed to liquid smoke at 2 and 4% concentrations in semi-moist pet food represent the times required for a 10-fold (1.0 log or 90%) destruction of the initial viable population of the mold. The decreases in *D*-values for the smoke treatments at 2 and 4% indicate a faster death rate of *A. flavus* at these concentrations in comparison to the untreated which did not have mold count reductions. The *D*-values also indicated faster death rate of *A. flavus* at 4% smoke concentration when compared to 2%. Even though *D*-values could not be obtained for several of the smoke treatments at 2% due to no consistent log reductions of mold count over time, it showed fungistatic effect of the liquid smoke inhibiting the mold growth.

Most of the pathogenic bacteria stop growing at water activities less than 0.87 while the growth of common spoilage yeasts and molds stops at 0.70 a_w_. Only xerophilic and osmophilic organisms can grow below 0.70 a_w_ and all microbial growth stops at water activities <0.60 (Scott, 1957; Beuchat, 1983). For a food product to be considered shelf stable, its water activity must be less than 0.86 to ensure that no pathogenic bacteria will be able to grow on the product as it sits on the shelf. Foods with a water activity higher than 0.70 but less than 0.86 are considered unfavorable for bacterial growth but will still support the growth of mold and yeast which affects the shelf-life stability of the product (Scott, 1957; Beuchat, 1983). Accordingly, semi-moist pet food products with a typical water activity of 0.65–0.70 require a_w_ to be controlled using humectants like polyols and to prevent mold growth require the use of preservatives like potassium sorbate at 0.1–0.3%. In our study, the experimental semi-moist pet food that we manufactured and tested had a water activity of 0.76 which is higher than normal, therefore we expect to see a higher fungistatic or fungicidal effect compared to that in this study when liquid smoke is applied to commercially manufactured semi-moist pet foods. Because consumers do not prefer having synthetic preservatives in their foods or pet foods for reasons described earlier, we suggest the use of liquid smoke as a natural preservative in semi-moist pet food.

The primary intention of smoking foods using a liquid form is to induce both a sought-after flavor and preservative effect. In addition, there may be microbiological effects of the applied smoke condensates. Liquid smoke is recognized as a GRAS (Generally Recognized as Safe) substance. This classification means that it can be used by food manufacturers without the need for a pre-market review verifying its safety. Furthermore, the use of GRAS substances is permitted as long as it is used in accordance with the manufacturers’ Good Manufacturing Practices (GMPs) (Rivera et al., 2021). Advantages and benefits associated with the use of liquid smoke in foods include ease and consistency of application to optimize antioxidant potential, sensory properties, and antimicrobial properties. Liquid smoke preparations can be easily controlled and evaluated for composition and consistency of application. Using condensates for smoke application allows the processor to dictate the concentration of smoke being applied more readily than using gaseous smoke (Sunen et al., 2001). The applied smoke can be evaluated for flavor acceptability in the product to determine the most suitable concentration. The antioxidant potential of smoke condensates has also been extensively documented, and its potential to retard lipid oxidation in many meat products is an added benefit (Maga, 1988; Estrada-Munoz et al., 1998). Another study by Deliephan (2022) reported that liquid smoke preparations containing medium to high carbonyl content repelled mites, thus aiding in mitigating their infestation in semi moist pet foods during storage. Due to these reasons, liquid smoke can be readily used in semi-moist pet food as well as other intermediate moisture food applications.

It can be concluded from the results of our study that liquid smoke preparations which had high to medium carbonyl content, namely, Cloud S-C100 and Cloud S-AC15 at concentrations of 2 and 4% (w/w) inclusion in semi-moist pet food can have fungicidal or fungistatic effects against storage molds like *A. flavus*. It can potentially be used as a substitute for synthetic mold inhibitors like potassium sorbate to some extent. We believe that liquid smoke inclusion at 2% in pet food can be a more feasible option compared to the 4%, which might be too high of a level for addition as a flavor component though this needs further corroboration. The usage levels of liquid smoke in food products like processed meat as reported by Lingbeck et al. (2014) ranged from 0.2 to 10% depending on the formula of liquid smoke. We could not find palatability evaluation results from previous published studies for usage levels of these liquid smoke preparations in pet food. For future work, we propose that the liquid smoke concentration levels in semi-moist pet food should be evaluated in palatability tests, for example, a two-bowl forced choice evaluation test for pets for practical applications. Spore counts for the molds were not performed in this study and we propose to include it in future work. We used the number of days to visible mold growth for shelf-life indication in this study. Visible mold growth is a qualitative technique, and we suggest performing mold counts as a quantitative method to correlate with shelf life in future experiments. Additionally, we believe that understanding the differences in formulation of these liquid smoke preparations by analyzing their compositions further using chromatographic analytical techniques could help explain differences in their antimycotic activity.

## Data availability statement

The original contributions presented in this study are included in the article/supplementary material, further inquiries can be directed to the corresponding author.

## Author contributions

AD contributed to the conceptualization, data gathering, analysis, and writing—original draft preparation. JD contributed to the conceptualization, supervision, and funding acquisition. BS contributed to the conceptualization, supervision, and writing—review and editing of original draft. CA contributed to the conceptualization, supervision, funding acquisition, and writing—review and editing of original draft. All authors read and approved the submitted version of the manuscript.
